# Supplementary material for: Ultrasound combined with urokinase under key-shaped bone window enhances blood clot lysis in an in vitro model of spontaneous intracerebral hemorrhage
Source: PLoS One. 2024 May 30;19(5):e0304398. doi: 10.1371/journal.pone.0304398 (PMC11139286; doi:10.1371/journal.pone.0304398)
Supplement: Attachment — Submitted filename: Response to Reviewers.docx [file pone.0304398.s002.docx]

Reviewer #1: nice work

although this work needs further validation by animal studies, it would be great if you mention the exact strength (other characteristics like frequency, power, duration) of ultrasound waves used for clot lysis.

Reply: Thank you for your comments and suggestions. We have provided the relevant characteristics of ultrasound wave. (page 5)

Reviewer #2: Cui et al are presenting a study aiming to evaluate the role of ultrasound combined with urokinase to enhance blood clot lysis in an in vitro model of spontaneous intracerebral hemorrhage. The manuscript is of scientific interest and contains data that can easily be translated to the clinical field. My major concern is the absence of discussion about the optimal frequency/duration and the absence of an explanation of the study sample calculation.

Reply: Thank you for your comments and suggestions.

We have provided an explanation of the study sample calculation. (page 5/6)

Since we only used one frequency and duration of ultrasound waves, we do not have sufficient data to support the discussion of frequency and duration. We have included this as a limitation of our study. We appreciate your understanding. (page 13)

I also have the following comments:

1. "Spontaneous intracerebral hemorrhage is classified as a subtype of stroke"- this information is trivial. Please remove this sentence.

Reply: Thank you for your comments and suggestions. We have made the appropriate revisions. (page 3)

2. "Reference 12 reported that ultrasound catheters combined with alteplase enhanced blood clot lysis in vitro" - please clarify the meaning of this sentence.

Reply: Thank you for your comments and suggestions. We have made the appropriate revisions. (page 4)

3. "However, due to the invasive nature of ultrasound catheters, their clinical application is currently restricted to peripheral vascular lesions" - provide the appropriate bibliographic support.

Reply: Thank you for your comments and suggestions. We have made the appropriate revisions. (page 4)

4. It is very hard to understand the meaning of figure 2 in the context of this study (in vitro experiment) - it is merely speculative.

Reply: Thank you for your comments and suggestions. We have revised the legends for Figure 1 and Figure 2. (page 16)

5. The manuscript needs English revision - some sentences are very difficult to understand.

Reply: Thank you for your comments and suggestions. We have made the appropriate revisions.
